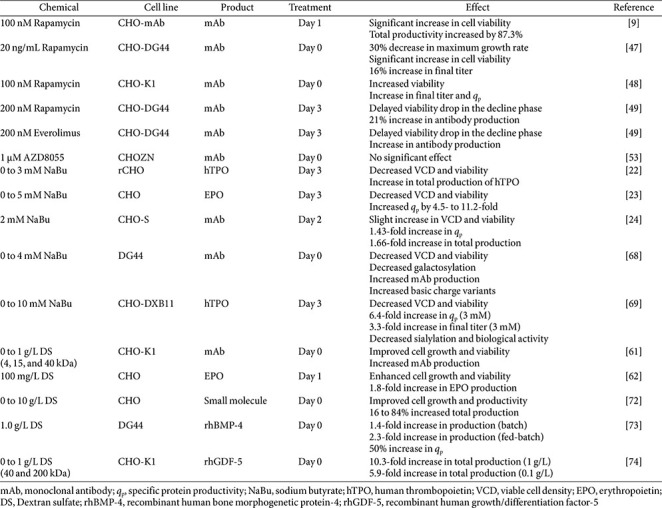# Chemicals Affecting Autophagy in rCHO Cell Cultures and Their Impacts on Therapeutic Protein Production Processes

**DOI:** 10.4014/jmb.2601.01037

**Published:** 2026-04-27

**Authors:** Hoon-Min Lee, Yeon-Gu Kim

**Affiliations:** 1 Biotherapeutics Translational Research Center, Korea Research Institute of Bioscience and Biotechnology (KRIBB), Daejeon 34141, Republic of Korea; 2 Department of Bioprocess Engineering, KRIBB School of Biotechnology, University of Science and Technology (UST), Daejeon 34113, Republic of Korea

**Keywords:** Recombinant CHO cell culture, Therapeutic protein production, Autophagy-inhibiting chemicals, Autophagy-inducing chemicals, Protein productivity

## Abstract

Autophagy is a cellular process that is essential for maintaining homeostasis in mammalian cells by degrading and recycling intracellular components, particularly under stress conditions. To achieve high therapeutic protein production in recombinant Chinese hamster ovary (rCHO) cells, it is necessary to develop a fed-batch culture process using optimized culture media that maintains a rapid growth rate and extends the culture longevity of cell lines with high specific protein productivity. During the development of these culture processes, stressful conditions, such as rapid nutrient deprivation and hyperosmolarity, which may occur through the repeated addition of nutrient concentrates, can induce autophagy in rCHO cells. Proper modulation of autophagy promotes cell survival by alleviating cellular stress rather than promoting cell death, thereby improving cell viability and therapeutic protein production in rCHO cell-based processes. This review discusses the various chemicals known to inhibit or induce autophagy in rCHO cell cultures, their mechanisms of action, and their effects on the production efficiency of therapeutic proteins. Understanding these mechanisms provides insight into improving the productivity and quality of therapeutic proteins during rCHO cell-based bioprocesses.

## Introduction

Recombinant Chinese hamster ovary (rCHO) cells are the most widely used workhorse for the production of recombinant therapeutic proteins, including monoclonal antibodies (mAbs), Fc-fusion glycoproteins, hormones, and cytokines. Their popularity stems from a number of advantages, including their high-efficiency protein expression system, their adaptability to large-scale bioreactors at high cell density, and ability to produce human-like glycans [1, 2]. However, maintaining long-term cell viability and high protein productivity during large-scale rCHO cell culture remains a significant challenge.

One of the major challenges in rCHO cell culture is managing programmed cell death (PCD), particularly apoptosis and autophagy, which affects cell viability, protein productivity, and protein quality during culture. Unlike apoptosis, which irreversibly induces cell death, autophagy is intricately involved in cell survival and death, which are dependent on the level of stress [3]. It can serve as a pro-survival mechanism that degrades and recycles damaged components to maintain energy levels and homeostasis under stressful conditions [4]. This helps maintain cell viability and alleviate cellular stress, ensuring sustained cell growth and delaying cell death. However, excessive or dysregulated autophagy may result in autophagic cell death [5]. Therefore, it is important to understand how this process is regulated in rCHO cells to optimize the production of therapeutic proteins.

Recently, autophagy has emerged as one of the important intracellular events in rCHO cell culture, affecting both cell viability and protein productivity. Many studies have increasingly focused on understanding the relationship between autophagy regulation and protein productivity through genetic engineering and/or chemical additives [6]. Various chemicals are known to either inhibit or induce autophagy, representing a potential tool for optimizing rCHO cell-based bioprocesses to produce therapeutic proteins. Modulating autophagy by various chemical treatments has a significant impact on both cell survival and recombinant protein production in rCHO cell cultures [7-9]; however, even with the use of the same chemicals during rCHO cell culture, the effect on autophagy can vary greatly depending on various factors, such as the concentration and timing of the treatment, specific cell line and culture medium, and type of recombinant protein being expressed. Therefore, identifying the specific intracellular mechanisms through which chemicals affect autophagy in rCHO cells and summarizing their effects is essential for using them in the therapeutic protein production process to achieve high production efficiency and product quality.

To date, there are no comprehensive reviews summarizing the various chemicals that regulate autophagy in rCHO cell culture and their impact on the therapeutic protein production process. This review addresses the different effects of the same compound by providing a detailed overview of the chemical compounds known to modulate autophagy in rCHO cells. It summarizes the mechanisms by which these chemicals act on autophagic pathways and their applications for improving rCHO cell-based bioprocesses for therapeutic protein production.

### Environments Affecting Autophagy in rCHO Cell Culture

Autophagy is a highly regulated cellular process that enables cells to adapt to environmental stresses by degrading and recycling intracellular components [5]. In rCHO cell culture, several conditions, including nutrient deprivation, hyperosmolality, and chemical additives, are known to induce autophagic responses. Autophagy in rCHO cells plays context-dependent roles, functioning as a pro-survival mechanism under moderate or transient stress conditions, while contributing to cell death when autophagic activity becomes excessive or prolonged under severe stress.

A primary trigger of autophagy during mammalian cell culture is nutrient deprivation. In rCHO cells, autophagy was first observed when essential nutrients, such as glucose and glutamine, were deficient toward the end of culture [10]. The results indicated that autophagy and apoptosis occurred simultaneously under nutrient deprivation conditions, as evidenced by the accumulation of microtubule-associated protein 1 light chain 3 (LC3)-II protein, a representative autophagic marker, and DNA fragmentation. Depletion of essential amino acids required for cell growth and metabolism similarly induces autophagy in rCHO cells [11], promoting metabolic adaptation to maintain mitochondrial function and ATP production under nutrient stress [12, 13]. Although autophagy also occurs in fed-batch cultures, its onset is delayed or attenuated compared with batch cultures without nutrient supplementation [14]. Overall, autophagy contributes to cellular homeostasis during nutrient limitation, thereby supporting prolonged cell survival and continued recombinant protein production.

Hyperosmolality is another major environmental stressor that induces autophagy in rCHO cells and is commonly encountered during fed-batch cultures due to repeated supplementation of nutrient concentrates and bases [15]. In mammalian cells, hyperosmolality negatively affects cell physiology, including reduced proliferation and altered glycosylation [16-18]. In rCHO cultures, sodium chloride-induced hyperosmolality increases autophagic flux, as evidenced by LC3 accumulation [19]. Unlike starvation-induced autophagy, hyperosmolality-induced autophagy occurs independently of the ULK1 complex [20]. Furthermore, Bcl-xL overexpression delays hyperosmolality-induced autophagic cell death by suppressing c-Jun *N*-terminal kinase (JNK) phosphorylation [21]. In this context, autophagy is considered a form of programmed cell death rather than a survival mechanism, as hyperosmotic stress primarily imposes structural rather than energetic challenges on cells.

In addition to environmental stresses, certain chemical additives used in rCHO cell culture can induce autophagy. Sodium butyrate (NaBu), a histone deacetylase (HDAC) inhibitor commonly applied to enhance specific protein productivity (*q*_p_), induces autophagy in rCHO cells in addition to its transcriptional effects [2, 22-24]. NaBu treatment has been shown to reduce mitochondrial membrane potential and promote the recruitment of mitophagy-related proteins, suggesting the selective removal of dysfunctional mitochondria. These observations indicate that NaBu-induced autophagy may function as a protective mechanism that supports cell survival by mitigating mitochondrial damage in rCHO cell cultures.

### Autophagy Pathways

**Fig. 1:**
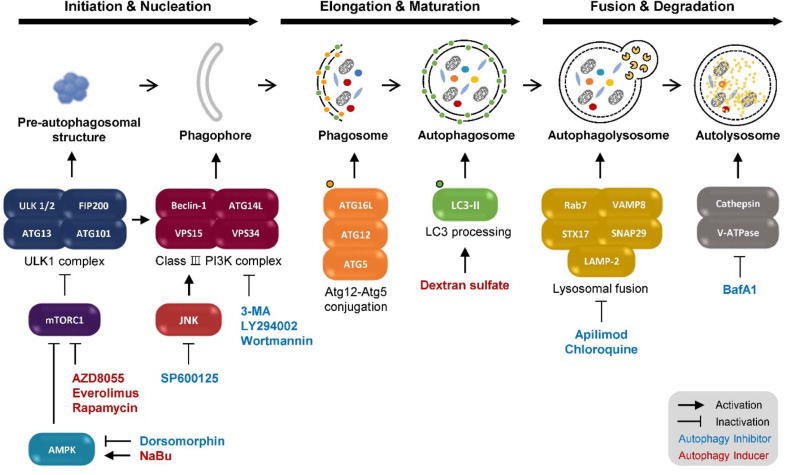
A schematic diagram of the autophagy process and chemicals in rCHO cells.

Autophagy is an evolutionarily conserved catabolic process in eukaryotic cells that enables the degradation and recycling of intracellular components in response to cellular stress. Upon stress sensing, a double-membraned autophagosome is formed, sequestering cytoplasmic cargo and delivering it to lysosomes for degradation [5]. This process proceeds through three sequential stages: initiation/nucleation, elongation/maturation, and fusion/degradation (Fig. 1).

**Initiation/Nucleation Stage.** Autophagy initiation is triggered by diverse cellular stresses, including nutrient deprivation, oxidative stress, and DNA damage. This stage is tightly regulated by energy-sensing pathways, particularly AMP-activated protein kinase (AMPK) and the mammalian target of rapamycin (mTOR), which together coordinate cellular metabolic status [25]. Under energy-depleted conditions, AMPK activation suppresses mTOR activity, leading to the activation of the Unc-51-like kinase 1 (ULK1) complex. Activated ULK1 promotes the assembly of autophagy-related proteins at the preautophagosomal structure, thereby initiating phagophore formation [26]. Phagophore nucleation is subsequently driven by the recruitment of the class III phosphatidylinositol 3-kinase (PI3K) complex, including Beclin-1, which generates phosphatidylinositol 3-phosphate to define membrane sites for autophagosome formation.

**Elongation/Maturation Stage.** Following nucleation, the phagophore expands and matures into a closed double-membraned autophagosome. This process is mediated by two ubiquitin-like conjugation systems: the Atg12-Atg5-Atg16L complex and the LC3 lipidation system [27]. The Atg12-Atg5-Atg16L complex facilitates phagophore membrane elongation, while LC3 is proteolytically processed and lipidated to form LC3-II, which is incorporated into the autophagosomal membrane [28]. LC3-II plays a critical role in membrane expansion, cargo recognition, and autophagosome maturation, and its accumulation is widely used as a marker of autophagic activity.

**Fusion/Degradation Stage.** In the final stage, mature autophagosomes fuse with lysosomes to form autolysosomes, enabling the degradation and recycling of sequestered cargo [29]. This process is coordinated by Rab7-mediated trafficking and soluble *N*-ethylmaleimide-sensitive factor attachment protein receptor-dependent membrane fusion, involving syntaxin 17 on autophagosomes and vesicle-associated membrane protein 8 on lysosomes, with synaptosomal-associated protein 29 acting as a bridging factor. Following fusion, lysosomal acidification by the vacuolar H^+^-ATPase (V-ATPase) activates hydrolytic enzymes, such as cathepsins, which degrade autophagosomal contents [30]. The resulting breakdown products are transported back to the cytoplasm and reused for cellular metabolism and biosynthesis.

### Autophagy-Inhibiting Chemicals in rCHO Cell Cultures

Autophagy in mammalian cells can be modulated by various chemicals that inhibit or induce this process through interactions with key signaling pathways and autophagy-related proteins. In rCHO cell cultures, several autophagy inhibitors have been identified and can be categorized according to the autophagic stage they affect, particularly the initiation/nucleation and fusion/degradation stages (Table 1). It should be noted that many chemical modulators of autophagy, although widely used as research tools, are not entirely specific and may exert off-target effects on other signaling pathways.

**Chemical Inhibitors at Initiation/Nucleation Stage.** During the early stages of autophagy, key regulatory molecules include the ULK1 complex and the class III PI3K complex (Fig. 1). Under stress conditions, autophagy is initiated through AMPK-mediated activation of the ULK1 complex following mTOR inhibition [31]. Chemical inhibition of these upstream regulators effectively suppresses autophagosome formation.

Dorsomorphin (compound C), a widely used AMPK inhibitor, suppresses autophagy initiation by preventing ULK1 phosphorylation [32]. This subsequently blocks the degradation and recycling of cellular components, resulting in reduced autophagic activity. In rCHO cells producing antibodies or Fc-fusion proteins, dorsomorphin treatment reduced autophagic activity, as confirmed by decreased LC3-II conversion and flow cytometry-based analyses [7]. SP600125, a JNK inhibitor, also suppresses autophagy by interfering with stress-induced activation of the class III PI3K complex [33]. JNK-mediated phosphorylation of Beclin-1 promotes its dissociation from anti-apoptotic proteins and facilitates PI3K complex activation [34]. In rCHO cell cultures, SP600125 treatment inhibited autophagy, as evidenced by altered LC3-II levels [7, 8].

Several inhibitors directly target the class III PI3K complex to block early autophagosome formation, including wortmannin, LY294002, and 3-methyladenine (3-MA) [35]. Both wortmannin and LY294002 inhibit PI3K kinase activity, suppressing phosphatidylinositol 3-phosphate production and thereby preventing phagophore and autophagosome formation. In rCHO cells, LY294002 effectively inhibited autophagy, as demonstrated by reduced accumulation of autophagic markers [7]. Although 3-MA is known to exert dual effects on autophagy depending on nutrient conditions, studies in rCHO cells consistently report reduced autophagic flux following 3-MA treatment, supporting the notion that it may be considered an autophagy inhibitor in this system [7, 36, 37].

**Inhibitors of Fusion/Degradation Stage.** The final stage of autophagy requires autophagosome-lysosome fusion and lysosomal acidification for cargo degradation. Bafilomycin A1 (BafA1) is a commonly used autophagy inhibitor that blocks lysosomal acidification by inhibiting the V-ATPase [38]. By inhibiting this proton pump, BafA1 prevents the lysosome from maintaining its acidic environment, which is essential for the activation of lysosomal enzymes required for the degradation of autophagic cargo. In rCHO cells, BafA1 treatment inhibits autophagy by increasing lysosomal pH and preventing autophagic degradation, leading to LC3-II accumulation [7, 8, 39]. Chloroquine is another widely used inhibitor of late-stage autophagy. Unlike BafA1, chloroquine primarily disrupts autophagosome-lysosome fusion rather than lysosomal acidification [40]. In rCHO cells, chloroquine impairs proper SNARE complex assembly during fusion, resulting in autophagosome accumulation and increased LC3-II levels [7, 41, 42].

Recent studies have identified apilimod, an inhibitor of FYVE finger-containing phosphoinositide kinase (PIKfyve), as a modulator of autophagy in rCHO cells [43]. Previous reports have demonstrated that apilimod increases autophagosome formation while suppressing lysosomal biogenesis through reduced expression of transcription factor EB, thereby inhibiting autophagosome-lysosome fusion. Because the fusion of autophagosome with lysosome is the final step in autophagy, apilimod can be considered an autophagy inhibitor based on the previous reports [44]. In addition, Gayle *et al*. (2017) proposed that reduced viability by apilimod is attributed to several factors, including the inhibition of lysosomal function and autophagy, which may lead to the accumulation of damaged cellular components and stress within the cells.

### Autophagy-Inducing Chemicals in rCHO Cell Cultures

Unlike autophagy-inhibiting chemicals, many autophagy-inducing compounds primarily activate the autophagy pathway at the initiation stage, most commonly through inhibition of the mammalian target of rapamycin (mTOR) pathway. However, several inducers promote autophagy through mTOR-independent mechanisms that are not yet fully understood. Representative autophagy-inducing chemicals in rCHO cell cultures are summarized below (Table 1).

**mTOR-Dependent Inducers.** Rapamycin is a representative autophagy inducer that is extensively used to study yeast and mammalian cells [45]. It induces autophagy by inhibiting mTOR signaling, thereby relieving suppression of the ULK1 complex and initiating autophagosome formation [46]. Multiple studies have demonstrated that rapamycin treatment induces autophagy in rCHO cell cultures, as evidenced by increased LC3-II levels and autophagosome formation [9, 47-49]. Moreover, rapamycin has been shown to extend culture longevity by maintaining high cell viability under nutrient-limited conditions.

Everolimus, also known as RAD001, is a rapamycin derivative that induces autophagy by inhibiting mTORC1 and promotes the formation of autophagosomes [50]. Both rapamycin and everolimus form a complex by binding to FK506-binding protein 12 and interact with the mTOR to inhibit the mTOR signaling pathway [51]. Although everolimus treatment in rCHO cells has not been studied as extensively as rapamycin, its treatment increased LC3-II expression, indicating autophagy induction via mechanisms comparable to those of rapamycin [49].

AZD8055 is another chemical that induces autophagy. Unlike rapamycin and everolimus, which mainly target mTORC1, this chemical inhibits both mTORC1 and mTORC2 [52]. Recent studies have begun to investigate the effects of AZD8055 on autophagy in antibody-producing rCHO cells, and AZD8055 has been shown to stimulate autophagy by inhibiting both mTORC1 and mTORC2 pathways [53]. Furthermore, similar to the effects of rapamycin, AZD8055 treatment led to increased cell viability, indicating a constructive response from cells resulting from autophagy stimulation.

**mTOR-Independent Inducers.** In addition to the mTORC1 signaling pathway, which is a classic autophagy pathway, autophagy can be induced through an mTOR-independent pathway [54]. NaBu, an HDAC inhibitor widely used in rCHO cultures, has been reported to induce autophagy in rCHO cells as well as in other mammalian cell types [22-24, 55, 56]. Several studies reported that this compound induces autophagy by activating AMPK, which in turn inhibits the mTOR signaling pathway [57, 58]. However, accumulating evidence suggests that this process is more closely associated with cellular stress responses, such as alterations in cytoplasmic calcium ion levels and endoplasmic reticulum (ER) stress, rather than canonical nutrient-sensing pathways that directly regulate the AMPK-mTOR pathway [56, 59]. Moreover, NaBu-induced autophagy has been associated with mitochondrial depolarization and the recruitment of mitophagy-related proteins, such as Parkin, suggesting a protective role of mitophagy in removing damaged mitochondria and supporting cellular homeostasis [23]. Given that these conditions are major pathways directly involved in autophagy and independent of mTOR, NaBu induces autophagy in an mTOR-independent manner in rCHO cell cultures, which may represent a response to stress in order to maintain homeostasis. Therefore, in this review, NaBu-induced autophagy in rCHO cell cultures is classified as predominantly mTOR-independent, as it appears to arise primarily from stress-mediated signaling mechanisms rather than direct modulation of nutrient-sensing pathways.

Dextran sulfate (DS), a sulfated polysaccharide commonly used as an anti-aggregation agent in rCHO suspension cultures, has also been reported to induce autophagy. This polysulfated compound can be produced in a variety of molecular weights, typically between 5 kDa and 500 kDa, and the molecular weight can affect its biological activity and efficacy in various applications [60, 61]. The treatment of this molecule promoted cell survival by delaying apoptosis and enhancing autophagic activity, as indicated by increased LC3-II conversion and autophagosome formation [62]. Although the mechanism underlying DS-induced autophagy remains poorly understood, it has been reported that DS supplementation induces cell cycle arrest at the G0/G1 phase in rCHO cell cultures [62]. Furthermore, a previous study suggested that autophagy is tightly regulated by the cell cycle, and cell cycle arrest at the G1 and/or S phases may contribute to autophagy activation [63]. In this context, the DS-induced autophagy may represent a protective response associated with cell cycle arrest, helping mitigate cellular stress and prevent apoptosis. In addition, DS treatment has been associated with increased expression of heat shock cognate protein 70, a key molecule of chaperone-mediated autophagy (CMA) [62]. This observation suggests that DS-induced autophagy, at least partially, involves CMA-related processes, although further research is necessary to clarify this mechanism. Consistent with its combined anti-apoptotic and pro-autophagic effects, DS-treated rCHO cells exhibit improved cell viability compared with untreated cultures, suggesting that DS may function as an autophagy-inducing agent that supports cell survival.

### Impact of Autophagy-Related Chemicals on the Production Process

Autophagy-related chemicals exhibit diverse effects on rCHO cell growth, viability, and therapeutic protein production, depending on both the stage of autophagy they target and the cellular conditions in which they are applied. In general, modulation of autophagy influences not only *q*_p_ but also culture longevity and protein quality attributes, highlighting the importance of carefully balancing autophagic activity during bioprocess development (Tables 2 and 3). The effects of autophagy-related chemicals described here are highly dependent on cell line characteristics, culture conditions, and the type of recombinant protein expressed, and therefore should not be generalized across all rCHO cell systems.

**Effect of Autophagy-Inhibiting Chemicals on Protein Production.** Autophagy-inhibiting chemicals that act at the initiation or nucleation stage, including dorsomorphin, SP600125, wortmannin, LY294002, and 3-methyladenine (3-MA), generally decrease cell growth and viability, while frequently enhancing *q*_p_. This trade-off between reduced cell proliferation and increased *q*_p_ has been consistently observed across multiple rCHO cell lines producing Fc-fusion glycoproteins, monoclonal antibodies, and other recombinant proteins [7, 8]. Although modulation of autophagy is closely associated with changes in *q*_p_, the contribution of secondary effects, such as cell cycle arrest and metabolic redistribution, cannot be excluded.

Inhibition of early-stage autophagy using dorsomorphin or SP600125 has been reported to result in significant decreases in viable cell density, yet yielded up to 1.5-1.6-fold increases in *q*_p_, suggesting a redistribution of cellular resources from cell proliferation toward recombinant protein synthesis. Similar trends were also observed with 3-MA treatment, which consistently enhanced *q*_p_ regardless of protein type or host cell line. Notably, optimized timing and dosing of 3-MA has been associated with marked improvements in both *q*_p_ and final product titer without adversely affecting *N*-glycosylation, highlighting its potential utility as a process yield enhancer [37, 64].

However, inhibition of autophagy at early stages can negatively impact protein quality under certain conditions. SP600125 and LY294002 treatments impaired *N*-glycosylation, particularly sialylation, of Fc-fusion glycoproteins. This effect was not attributed to altered expression of glycosylation-related genes, but was associated with reduced availability of intracellular nucleotide sugars, suggesting that autophagy inhibition may disrupt metabolic precursor recycling required for optimal glycan processing [8].

In contrast, autophagy inhibitors targeting the fusion/degradation stage, such as BafA1 and chloroquine, generally exhibited limited positive effects on *q*_p_. Moreover, BafA1 treatment also significantly reduced sialylation of glycoproteins, likely due to impaired lysosomal function and altered Golgi pH, which can indirectly affect glycosylation enzyme activity. Although chloroquine inhibits late-stage autophagy through a similar mechanism with BafA1, its impact on cell growth, viability, and *q*_p_ is less apparent in rCHO cultures [7]. Apilimod, which disrupts autophagic flux at the fusion stage, similarly enhanced *q*_p_ despite reduced cell growth due to cell cycle arrest at the G0/G1 phase, supporting the correlation between proliferation control and increased productivity [43].

Overall, autophagy-inhibiting chemicals may enhance *q*_p_ along with frequently inducing adverse effects on product quality, particularly reduced sialylation. Although the current review primarily focuses on sialylation as an indicator of product quality, it should be noted that critical quality attributes (CQAs) of therapeutic proteins encompass other attributes, such as high-mannose species, core fucosylation, and charge variants. To date, these attributes have not been systematically investigated in the context of autophagy inhibition, and relevant studies remain limited. One possible hypothesis for the observed changes in sialylation is that increased *q*_p_ indirectly affects glycan maturation. In fact, a previous study reported that increased *q*_p_ in CHO cells resulted in reduced galactosylation of mAbs, potentially due to the shortened residence time of proteins in the Golgi apparatus, thereby limiting glycan processing [65]. In addition to increased *q*_p_, Golgi pH is known to play a critical role in glycan maturation, and an elevated Golgi pH may further affect the localization and activity of glycosyltransferases. In particular, the addition of BafA1 can cause changes in Golgi pH and result in immature *N*-glycans not only in CHO cells but also in other mammalian cells [66, 67]. Taken together, although current evidence suggests that autophagy inhibition can influence glycosylation, a comprehensive investigation of its effect on a broader range of CQAs is still lacking. Therefore, the application of autophagy inhibitors to increase *q*_p_ must be carefully considered, particularly with respect to potential effects on product quality.

**Effect of Autophagy-Inducing Chemicals on Protein Production.** Autophagy-inducing chemicals influence rCHO cell cultures in a manner distinct from autophagy inhibitors, with outcomes strongly dependent on whether induction occurs via mTOR-dependent or -independent pathways. mTOR-dependent inducers, such as rapamycin and everolimus, consistently improve culture longevity by delaying viability loss during the death phase, thereby increasing the integral of viable cell density and final product titer.

Rapamycin treatment across multiple rCHO cell lines resulted in modest reductions in growth rate but significant increases in overall protein production, driven primarily by prolonged cell viability. Regardless of the concentration, timing, and type of protein product produced, treatment with rapamycin delayed the loss of cell viability, resulting in an increase in volumetric productivity [9, 47-49]. Similar effects were observed with everolimus, supporting the concept that controlled mTOR inhibition promotes a favorable balance between survival and productivity. Importantly, although studies related to protein quality are still limited, findings suggest that rapamycin does not significantly impair sialylation of glycoproteins in early culture stages of rCHO cells [8].

In contrast, mTOR-independent autophagy inducers exhibit more variable effects in rCHO cell cultures. NaBu is an mTOR-independent inducer used in rCHO cell culture and is known as a *q*_p_-enhancing agent for various therapeutic protein production, such as human thrombopoietin (hTPO), EPO, and mAb [23, 68, 69]. In contrast to the positive effects of rapamycin treatment in rCHO cells, NaBu did not improve the integral of viable cell density and did not ensure an increase in total protein production despite the increase in *q*_p_. Although the mechanism by which NaBu-induced autophagy increases *q*_p_ has not yet been clearly elucidated, a possible explanation is that it contributes to maintaining ATP supply and cellular homeostasis under NaBu-induced stress by removing dysfunctional mitochondria and damaged components through mitophagy [70]. Although few reports have described the effects of mTOR-dependent autophagy-inducing chemical treatments on protein quality, numerous studies have examined the effects of NaBu. Increasing concentrations of NaBu significantly impaired protein quality, including acidic isoforms, sialylation and galactosylation [68, 69]. In rCHO cell culture, protein impairment associated with NaBu is mainly caused by the release of intracellular proteases along with membrane disruption resulting from decreased cell viability [69]. Another reason may be the alteration of *N*-glycosylation-related gene expression, as evidenced by the low protein quality following NaBu treatment in cell samples with viability > 95% [71].

DS is another potential autophagy-inducing chemical that acts in an mTOR-independent manner in rCHO cells. Menvielle *et al*. (2013) reported that 100 mg/L DS supplementation enhanced cell growth and protein production of EPO-producing rCHO cells acting as a pro-autophagic agent [62]. Two studies dealing with DS addition to rCHO cells producing small protein < 75 kDa and mAb indicated that it also improved therapeutic protein production by enhanced cell growth, although they did not demonstrate its autophagy-inducing effectiveness [61, 72]. Besides the positive effect of DS on cell growth, DS enhanced *q*_p_ and final product titer in rCHO cells producing recombinant human bone morphogenetic protein-4 and recombinant human growth/differentiation factor-5 by blocking re-internalization of produced protein [73, 74]. Similar to NaBu, the mechanistic basis of DS-induced autophagy requires further investigation. However, considering the observed upregulation of CMA-related proteins after DS supplementation, it is possible that CMA, which can be activated under stress conditions such as nutrient limitation and oxidative stress and facilitates the recycling of intracellular proteins to supply amino acids, may help alleviate cellular stress and thereby indirectly contribute to increased *q*_p_ [75-77]. However, studies addressing the impact of autophagy-inducing chemicals on product quality remain limited, highlighting the need for further systematic investigation.

Similar to that of autophagy inhibitors, the investigation of product quality in studies using autophagy-inducing chemicals has been largely limited to a few attributes, primarily galactosylation, sialylation, and, in some cases, charge variants, particularly in NaBu-treated cultures. Nevertheless, because the impact of sialylation on efficacy varies depending on the type of therapeutic protein, it may not always be considered a CQA [78]. Therefore, a broader investigation of additional quality attributes, including other glycosylation profiles, may be required under various autophagy-inducing conditions. Due to these limitations, it is difficult to determine whether the relatively mild effects observed with mTOR-dependent inducers, such as rapamycin, indicate a negligible impact on product quality or are a result of a lack of comprehensive investigation. In contrast, several cases of quality impairments associated with NaBu suggest that mTOR-independent induction exerts more pronounced effects; however, these observations could also have been affected by secondary factors such as reduced cell viability and altered cellular metabolism. Therefore, a more comprehensive and systematic investigation of glycosylation profiles and other CQAs will be required to clarify in detail the impact of autophagy induction strategies on the quality of therapeutic proteins.

## Conclusion

Autophagy is an essential process for cell survival in mammalian cells. It involves recycling damaged components and using them as substrates for energy production. Regulating autophagy using various chemicals to modulate therapeutic protein production in rCHO cells is considered a promising approach because it is relatively simple and straightforward. Some autophagy-inhibiting chemicals exhibit a positive effect in increasing the *q*_p_ of rCHO cells, but most have negative effects on viable cell density (Table 2). However, autophagy-inducing chemicals, particularly mTOR-dependent inducers, generally improved cell viability at the end of batch culture, thereby increasing overall protein production (Table 3). This suggests that chemically induced autophagy in rCHO cells is a beneficial mechanism for cell survival and can be exploited to efficiently produce therapeutic proteins in rCHO-based bioprocesses. In this review, we have discussed only a few autophagy-related chemicals used in rCHO cell culture; however, many other autophagy-inducing and -inhibiting chemicals are used in other mammalian cells or in cancer therapy [79]. Therefore, it is worthwhile to examine the effects of other autophagy-related chemicals in rCHO cell culture to explore new applications for the production process.

A key challenge when using chemicals in rCHO cell culture is the potential for off-target effects. In general, chemical modulators may unexpectedly interact with other signaling pathways due to their complexity, which may also depend on the amount and treatment time. It is important to recognize that the use of autophagy-related chemicals may also result in other unexpected and non-specific side effects. Indeed, rCHO cell-based studies have revealed that the same chemical treatment has different effects on cell growth and *q*_p_ depending on the timing, concentration, and type of cell line used. Therefore, to ensure versatility, combining autophagy-related chemical additives with genetic engineering techniques targeting various autophagy-related genes may be a potent strategy. Furthermore, the comprehensive characterization of rCHO cells through multi-omics analysis based on systems biology has recently attracted attention [80, 81]. It will be beneficial to thoroughly analyze the transcriptomic and metabolomic changes in rCHO cells following treatment with autophagy-inducing and autophagy-inhibiting chemicals.

Besides its effectiveness on therapeutic protein production, autophagy also functions as a protective mechanism against ER stress and may also contribute to protein quality in rCHO cell culture. The UPR activates autophagy to relieve ER stress caused by misfolded protein accumulation in rCHO cell culture [82]. Dysfunctional autophagy results in increased ER stress due to the failure of selective degradation of misfolded proteins. Elevated ER stress in rCHO cells leads to decreased secretion of recombinant proteins, increased aggregation, and altered glycosylation patterns [83, 84]. Based on the close relationship between autophagy and ER stress, future studies on ER stress modulation using autophagy-related chemicals will improve not only protein production, but also protein quality in rCHO cell cultures.

In conclusion, although the potential of autophagy regulation to improve recombinant protein production in rCHO cells is promising, current understanding remains limited due to incomplete mechanistic clarification of the relationship between autophagy, protein productivity, and product quality. Therefore, future studies must focus on elucidating the underlying mechanisms linking autophagy to cellular metabolism, protein synthesis, and quality control processes. Moreover, culture process optimization will be essential to completely exploit autophagy-modulating strategies, particularly with respect to the timing, dosage, and duration of chemical treatment in dynamic culture environments. A more systematic investigation into the relationship between chemical modulation and CQAs, including glycosylation profiles, charge variants, and protein aggregation, will also be necessary to ensure both productivity and product quality. In addition, integrating autophagy-related chemical approaches with targeted genetic engineering strategies may enable more precise and robust control of autophagic flux and its downstream effects, ultimately leading to efficient improvements in rCHO cell-based bioprocesses.

## Figures and Tables

**Table 1:** Autophagy-regulating chemicals used in rCHO cell cultures

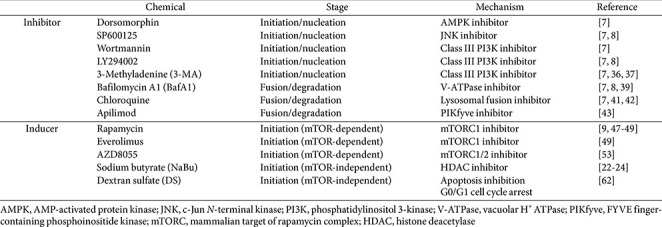

**Table 2:** Effects of autophagy-inhibiting chemicals on therapeutic protein production in rCHO cell cultures

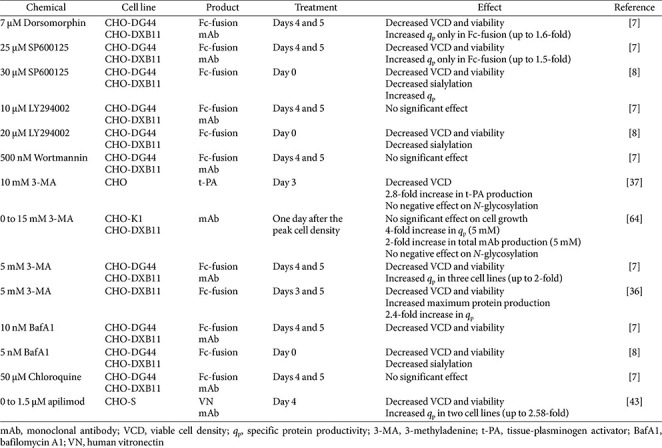

**Table 3:** Effects of autophagy-inducing chemicals on therapeutic protein production in rCHO cell culture